# All-nanotube stretchable supercapacitor with low equivalent series resistance

**DOI:** 10.1038/s41598-017-17801-4

**Published:** 2017-12-12

**Authors:** Evgenia P. Gilshteyn, Daler Amanbayev, Anton S. Anisimov, Tanja Kallio, Albert G. Nasibulin

**Affiliations:** 1 Skolkovo Institute of Science and Technology, Laboratory of Nanomaterials, Nobel str. 3, Skolkovo, Moscow 143025 Russia; 2 Canatu Ltd., Konalankuja 5, FI-00390 Helsinki, Finland; 3 Research Group of Electrochemical Energy Conversion and Storage, Department of Chemistry and Material Science, School of Chemical Engeneering, Aalto University, P.O. Box 16100, FI-00076 Aalto, Finland; 4 Department of Applied Physics, School of Science, Aalto University, P.O. Box 15100, FI-00076 Aalto, Finland

## Abstract

We report high-performance, stable, low equivalent series resistance all-nanotube stretchable supercapacitor based on single-walled carbon nanotube film electrodes and a boron nitride nanotube separator. A layer of boron nitride nanotubes, fabricated by airbrushing from isopropanol dispersion, allows avoiding problem of high internal resistance and short-circuiting of supercapacitors. The device, fabricated in a two-electrode test cell configuration, demonstrates electrochemical double layer capacitance mechanism and retains 96% of its initial capacitance after 20 000 electrochemical charging/discharging cycles with the specific capacitance value of 82 F g^−1^ and low equivalent series resistance of 4.6 Ω. The stretchable supercapacitor prototype withstands at least 1000 cycles of 50% strain with a slight increase in the volumetric capacitance from 0.4 to 0.5 mF cm^−3^ and volumetric power density from 32 mW cm^−3^ to 40 mW cm^−3^ after stretching, which is higher than reported before. Moreover, a low resistance of 250 Ω for the as-fabricated stretchable prototype was obtained, which slightly decreased with the strain applied up to 200 Ω. Simple fabrication process of such devices can be easily extended making the all-nanotube stretchable supercapacitors, presented here, promising elements in future wearable devices.

## Introduction

Currently, research in the domain of flexible and stretchable supercapacitors is focused on adjusting electrodes, as they affect the performance the most^1–3^. However, the separator materials for such applications are left relatively unexplored. Besides being dielectric, porous and chemically inert, the separators for stretchable supercapacitors need to withstand multiple bending and stretching without severe structural damages. The materials that meet afore-mentioned requirements are polymers and polymer-based electrolytes, i.e. polyurethane membranes and polyvinyl alcohol (PVA)-based electrolytes^4,5^. However, despite been inexpensive, non-toxic and highly stretchable, polyurethane membranes made by electrospinning are thicker than any other separator materials (0.2 mm). Other polymer separators (polypropylene, polyethylene), which are normally used in liquid electrolyte systems, offer several advantages, such as good chemical stability, simplicity of manufacturing and processing. However, there are several drawbacks, which are still present and cannot be easily solved. The polymer separators show poor wetting with aqueous electrolytes. Their thicknesses are usually above 20 μm and the attempts to create thinner polymer films usually reveal the problems with their mechanical strength, and as a result they are unable to ensure reliable short circuit protection. As such separators are not appropriate for stretchable supercapacitor applications, another type of polyvinyl alcohol based electrolytes has been already investigated, which additionally play role of separators and gluing materials and can be stretchable after solidification. The smallest thickness of PVA separator is reported to be 150 μm^6^, resulting in relatively high internal resistances. In contrast with separator materials mentioned above, some nanomaterials show outstanding mechanical properties being less than 1 μm thick^7,8^. In particular, boron nitride nanotubes (BNNTs) is a dielectric nanomaterial that shows high Young’s modulus and tensile strength^9^. Generally, the BNNT film is a catalyst-free, dielectric^10,11^, entangled and porous material, chemically inert in strong acids and alkalis^12^, composed of incredibly strong individual BNNTs, and thus considered perfect materials for separator applications. So far very few works have been published where the BNNTs utilised as the separator^13,14^. None of them was either flexible or stretchable. However, due to its remarkable properties, BNNTs are believed to be able to fulfil the requirements of the growing industry and to provide a reliable and stable separator for stretchable supercapacitors.

Another key component of the supercapacitors are electrodes, which could be made of carbon nanotube films (CNTs) due to their unique pore structure, narrow distribution size, high specific surface area, low electrical resistivity and high chemical stability^15,16^. Moreover, single-walled carbon nanotubes (SWCNTs) possess many unique properties, which are advantageous for a wide variety of applications, including stretchable electronics^17^. They have exceptionally high Young’s modulus of elasticity and tensile strength and are one of the strongest known material^18^. The porosity and specific surface area of SWCNT films are very large, and they possess high transparency and flexibility^19^. In addition, SWCNTs can withstand extremely high current densities (up to 10^9^ A cm^−2^) making them an ideal replacement for copper and aluminium in fast integrated charge/discharge circuits^20^.

In this work, we applied thin films of SWCNTs as the electrodes and BNNTs as the separator to fabricate all-nanotube stretchable supercapacitors. The SWCNTs and BNNTs films were chosen to be used together due to several important qualities, such as costs, availability and their ideal structures for the use as an electrode and a separator, respectively. The lattice structures, which strengthen the material between walls of both materials, make it possible to test and characterize the device under mechanical stretching. We successfully solved the problem of separator thickness and resistance keeping elastic properties of the device. Remarkably, high specific capacitance and stability were reached with the SWCNT electrodes in test cells^21^ and they were successfully used to construct stretchable device^22,23^. The BNNT separator of only 0.5 µm thickness ensured reliable short circuit protection and low equivalent series resistance (ESR) of the stretchable supercapacitor (SSC). The stretchable all-nanotube supercapacitor prototype withstood at least 1000 cycles of 50% strain without significant changes in performance. The technology of the SSC fabrication is very simple, as it is based on dry deposition transferring and airbrushing techniques. With its stable performance, the device could act as a promising candidate for the wearable electronic devices and flexible energy storage systems.

## Experimental

### Materials and characterization

The SWCNT films were synthesized by an aerosol chemical vapour deposition (CVD) method described elsewhere^24,25^. Briefly, a vapour of catalyst precursor, ferrocene, is supplied into the CVD reactor, where it decomposes in the atmosphere of carbon monoxide, forms nanometer catalyst particles on the surface of which carbon monoxide (CO) disproportionation occurs and finally SWCNTs grow. The flow at the outlet of the rector is filtered and SWCNTs are collected onto the nitrocellulose filter. Simply, by varying the duration of the collection time one could obtain the films of different thickness and transparency. Importantly, the SWCNT films could be easily transferred to different substrates by dry-deposition technique^26,27^. BNNTs for the separator fabrication were provided by BNNT, LLC. Sulfuric acid (H_2_SO_4_) and polyvinyl alcohol powder were purchased from Sigma-Aldrich. Polydimethylsiloxane (PDMS) Sylgard 184 kit by Dow Corning Corp., consisted of elastomer base and curing agent were used.

The morphology characterization of the SWCNT films and BNNT samples was performed by FEI Versa 3D DualBeam scanning electron microscope with the acceleration voltage 30 kV, equipped by Focused Ion Beam (FIB) tool for materials deposition and cross sectioning. PANalytical X’Pert Pro MPD Alpha-1 X-Ray Diffractometer with K-alpha radiation (0.15406 nm) was used to characterize the structure and quality of the BNNTs. Raman spectrometer Horiba Jobin-Yvon Labram HR with a 633 nm laser was used to investigate the purity of the SWCNT films.

### Two-electrode cell and electrochemical measurements

**Figure 1:**
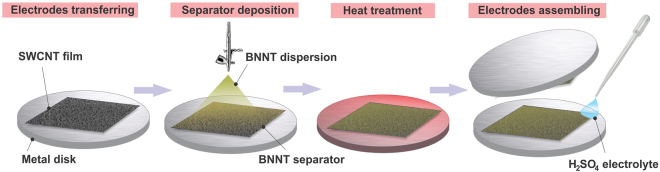
Schematic two-electrode cell assembly process.

We characterized the electrochemical properties of the materials using test cell (Hohsen Corp.) in two-electrode configuration. Figure 1 schematically shows the assembly procedure of the two-electrode test cell. The SWCNT films (1 × 1 cm^2^) were transferred by a simple dry-press technique^27^ to the top and bottom current collectors.

After this, BNNT dispersions were prepared according to procedure previously described^28^. 1 mg of BNNTs were sonicated in Elmasonic S30 (H) ultrasonic bath with 5 ml of toluene for 15–30 minutes yielding fine dispersion that in less than a minute precipitated and accumulated in the bottom of the vial. A few minutes later, 3 ml of toluene from the upper part of the vial were substituted by 3 ml of DMF with the use of micropipette. The resulting mixture was bath-sonicated for 1–2 minutes. Interestingly, the attempts to disperse BNNTs directly with the DMF/toluene mixture were not successful and produced unstable dispersion. The BNNT separator was deposited onto both SWCNT film electrodes so that it covered the electrode entirely by using airbrush to spread the 5 ml BNNT/toluene-DMF dispersion at 207 kPa with simultaneous heating of the current collectors at 80 °C. Then all the parts were thermally treated at 220 °С for 40 minutes. The thermal treatment is a crucial step as it removes the hydrocarbon residues from the SWCNT synthesis as well as toluene and DMF from the BNNT separator, and thus increases the electrochemical stability of the device. Subsequently, few drops (less than 0.5 ml) of 1 M H_2_SO_4_ liquid electrolyte were added on top of the BNNT separator, and both the top and bottom parts are screwed together (Fig. 1).

The electrochemical performance of the materials used in the two-electrode test cell was assessed with the use of cyclic voltammetry (CV), galvanostatic charge-discharge (GCD) and electrochemical impedance spectroscopy (EIS) methods using an Elins P-40X potentiostat-galvanostat. During the CV measurements, the as prepared cells were cycled at 200 mV s^−1^ for 50 times to stabilize the cell before the data was collected. Subsequently, the CV curves were recorded from 200 to 1000 mV s^−1^ scan rates with 100 cycles for each scan rate. For the scan rate of 200 mV s^−1^ the test cell was further tested for durability under 20 000 CV cycles. GCD measurements were performed at different DC currents throughout the entire potential range up to 25 A g^−1^. The EIS spectra of the two-electrode test cell have been collected in a potentiostatic mode with the 5 mV amplitude in the frequency range from 1 Hz to 500 kHz with 50 points in total.

### Fabrication of the all-nanotube SWCNT/BNNT stretchable supercapacitor prototype

**Figure 2:**
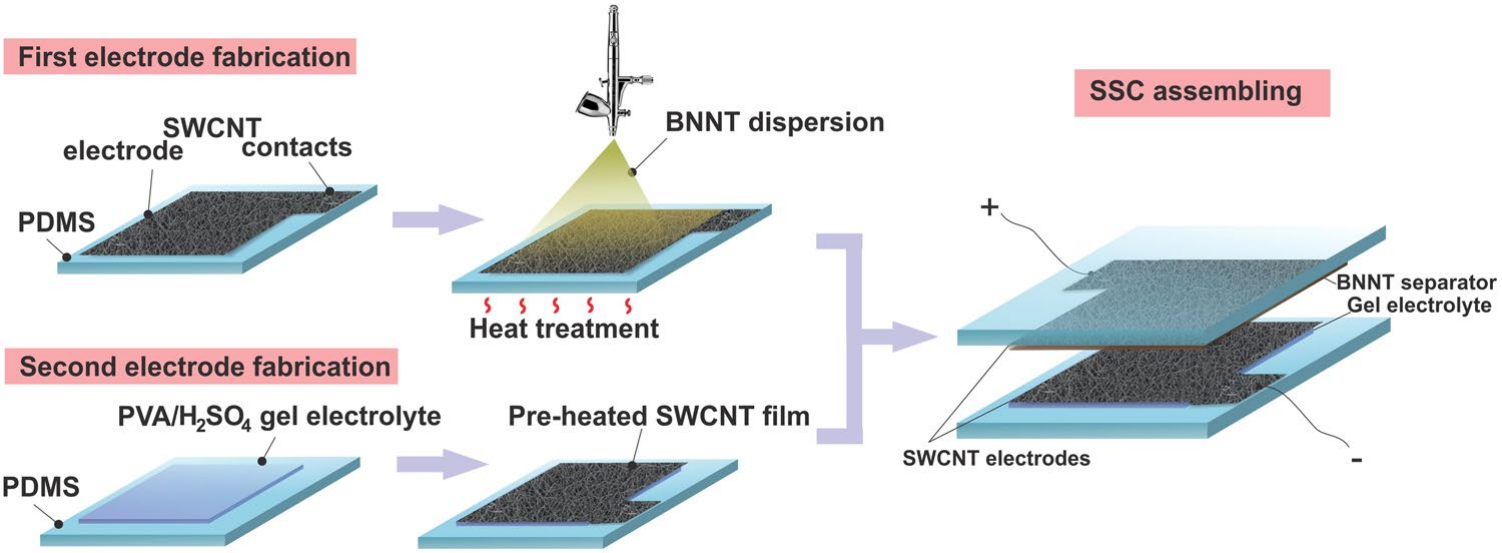
Process flow of stretchable supercapacitor fabrication.

For the stretchable prototype PDMS substrates were prepared. The base and curing agent were mixed with 10:1 ratio by mass, poured onto a laboratory glass, degassed and cured at 90 °C for 40 minutes. After the curing, the sample was cut into rectangular pieces of a desired size. The whole process of SSC assembly is schematically shown in Fig. 2. For the SSC prototype fabrication dimethylformamide (DMF) and toluene were replaced by isopropanol (IPA) to prepare the dispersion of BNNTs. This can be explained by the fact that the prototype cannot be heated to remove DMF and toluene from the SWCNT electrode surface and toluene causes swelling of the PDMS substrate. Similarly, BNNTs were sonicated in toluene. After the sonication, 3 cycles of centrifugation in Sigma 3**–**30KS centrifuge at 6000 RPM for 5 minutes were performed with the subsequent toluene supernatant removal after each step. This was followed by 3 cycles of washing the BNNTs with the equal volume of IPA and centrifugation under the same parameters. The final BNNT/IPA mixture was bath sonicated for 30 minutes yielding a fine dispersion.

After BNNT/IPA dispersion was prepared two electrodes were assembled as follows. The SWCNT film (2 × 1 cm^2^) was deposited on top of the first PDMS substrate and 0.5 ml BNNT separator drop-casted from the BNNT/IPA dispersion. The whole structure was heated up to 220 °C for 40 minutes. The second PDMS substrate was covered with a thin layer of a PVA/H_2_SO_4_ gel-electrolyte and SWCNT films were transferred on top of it. The need for PVA in the supercapacitor prototype is dictated by the fact, that under stretching conditions practically any liquid leaks from the device. To solve this problem, we utilized the solid-state electrolyte (PVA and H_2_SO_4_), proven to withstand the mechanical stresses without the electrolyte leakage^22^. It should be noted that the PVA/H_2_SO_4_ gel rapidly decomposes when heated, and thus the thermal treatment of the SWCNT films needs to be completed beforehand.

After the solvent evaporation, two semi-plates were attached facing each other and glued at the edges with PDMS and left to dry overnight. Here, H_2_SO_4_ plays the role of the electrolyte and impregnates the SWCNT electrodes, and the BNNTs act as a separator. Similar techniques, described above for the two-electrode cell, were used for investigation of the electrochemical performance of the fabricated stretchable supercapacitor (CV, GCD and EIS methods).

## Results and Discussion

**Figure 3:**
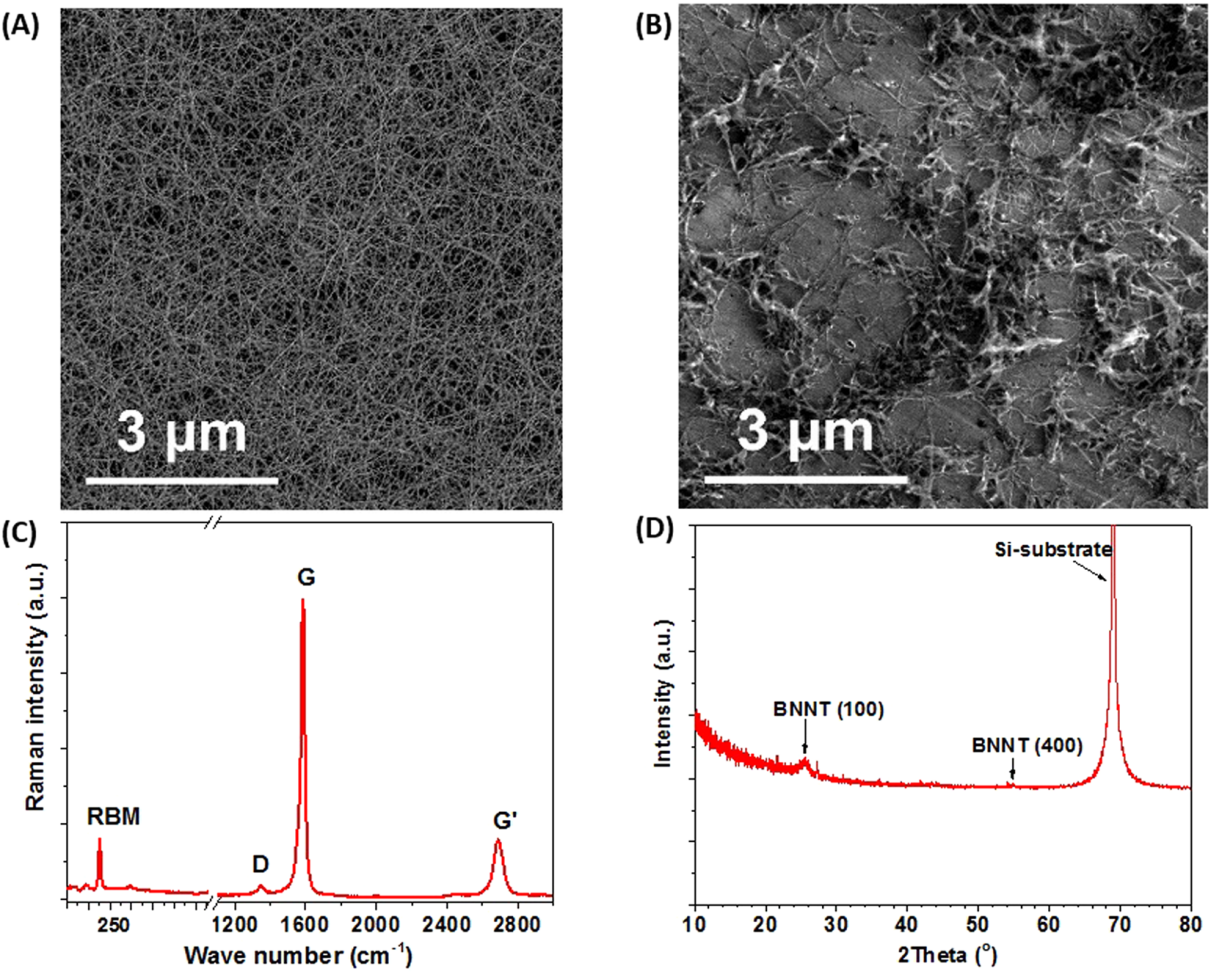
The characterization of materials. SEM micrographs of (**A**) the SWCNT film and (**B**) the BNNT film. (**C**) Raman spectrum of the SWCNT film and (**D**) XRD spectrum of pristine BNNTs.

Uniform structure of the aerosol synthesized SWCNT film could be confirmed based on SEM microscopy (Fig. 3A). Due to the entangled structure, the SWCNT films can be stretched without noticeable changes in electrical conductivity and moreover, they possess high specific surface area^29^, that makes them ideal for the electric double-layer capacitor (EDLC) electrode.

Raman spectrum of the tubes deposited on a quartz substrate demonstrates typical SWCNT spectrum (Fig. 3C). G-band around 1580 cm^−1^ is indicative of a graphitic structure, while D-band around 1350 cm^−1^ corresponds to disordered carbon. High intensity ratio between G and D bands (1:35) shows that the SWCNTs are of high quality that determines unique mechanical and electrical properties. For SEM imaging, pristine cotton-like BNNTs were put in contact with sticky conductive carbon layer. As could be seen from a SEM image of raw BNNTs (Fig. 3B), the sample initially comes as a network of bundled nanotubes with high aspect ratio. Darker spots stand for boron impurities that are not affecting the SSC performance. The structure of BNNTs was characterized by the means of XRD (Fig. 3D). (100) h-BN and (400) h-BN peaks around 28° and 56° are of high intensity in a pristine sample. Here, h-BN is a hexagonal BN that being rolled into a tube forms a BNNT, by analogy with the graphene and CNTs.

**Figure 4:**
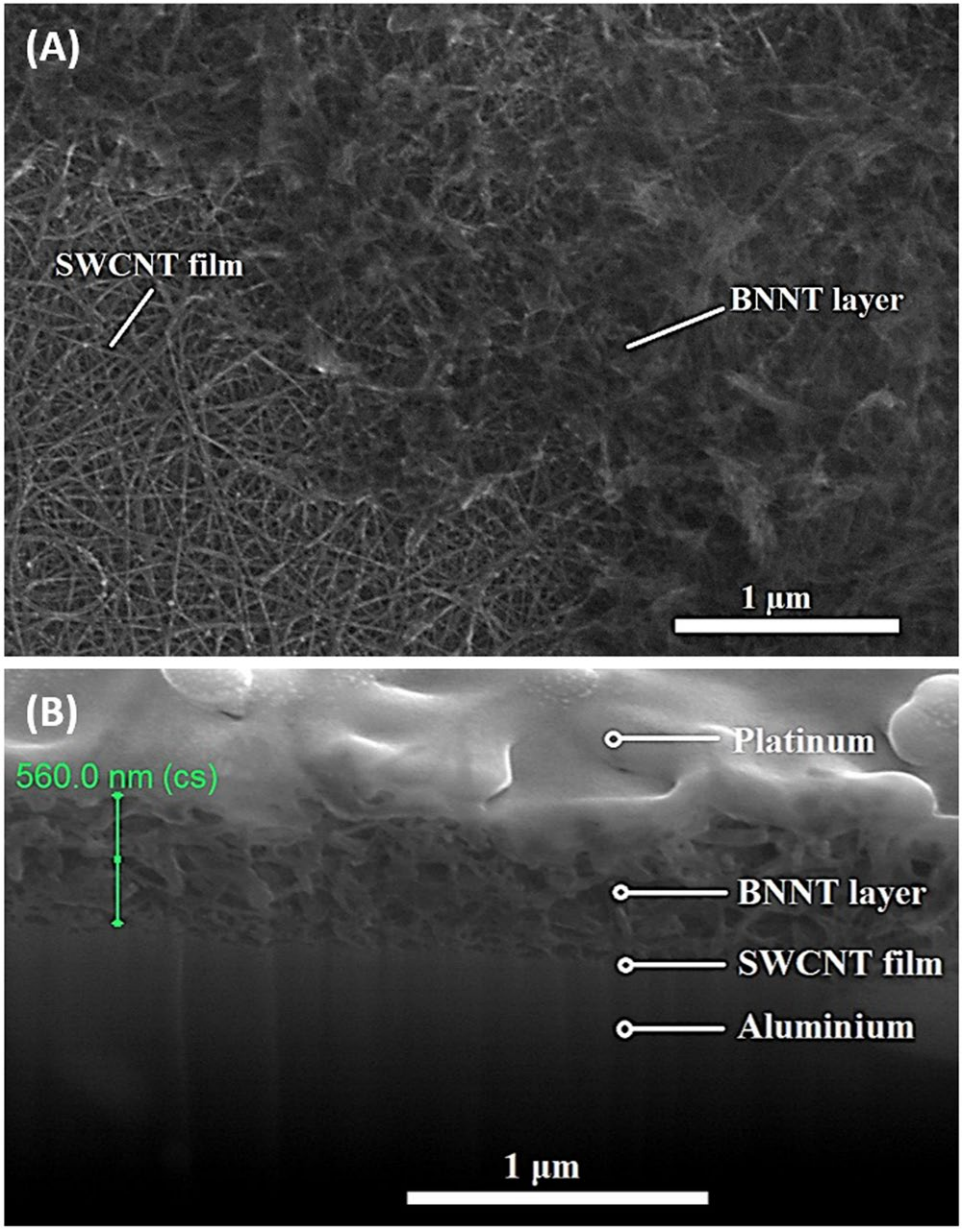
SEM images of the separator layer. (**A**) BNNT separator on top of the SWCNT film electrode on PDMS, (**B**) cross-section of the BNNT layer.

The first set of experiments was dedicated to the investigation of the materials’ performance in a two-electrode cell configuration. The assembly procedure of the test cell was described in paragraph 2.2 and schematically shown in Fig. 1. The morphology of the BNNT separator on top of the SWCNT film was characterized by SEM (Fig. 4A). The BNNT layer forms a porous structure that is beneficial to the SSC in terms of mass transport and ion permeability^30^. In order to estimate the thickness of the separator layer, BNNTs were deposited on the surface of the SWCNT film transferred on an aluminum foil and the cross-section of the structure was cut by means of SEM FIB. As can be seen from Fig. 4B the thickness of the BNNT films utilized for SSC fabrication was around 550 nm.

**Figure 5:**
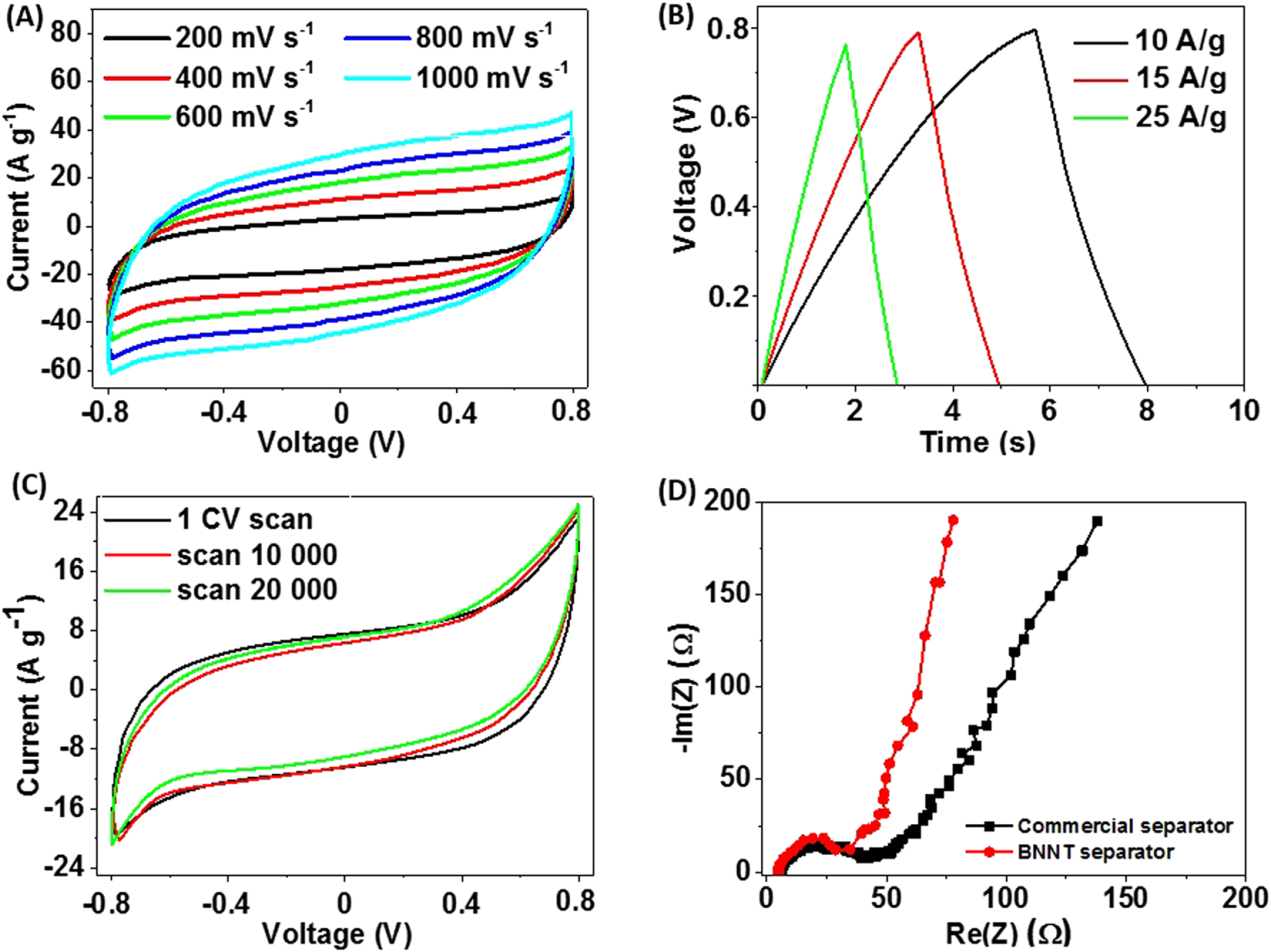
Electrochemical performance of the two-electrode test cell. (**A**) CV curves at various scan rates. (**B**) Galvanostatic charge-discharge at high currents. (**C**) CV curves of 20 000 load cycles collected at a scan rate of 200 mV s^−1^. (**D**) Electrochemical impedance spectra of the BNNT separator (blue) and commercial separator (black).

Almost rectangular shape of the CV curves (Fig. 5A) indicates EDLC capacitance of the cell being measured at 200, 400, 600, 800 and 1000 mV s^−1^ scan rates. Perfect shape of the CVs can be attributed to good transport properties of the BNNT separator. Specific capacitance by mass of the electrode *(C*_*sp*_) was calculated from the CV curves according to eqn (1)^31^:

1 $${{\boldsymbol{C}}}_{{\boldsymbol{sp}}}=\frac{1}{2{\boldsymbol{m}}{\rm{\Delta }}V{\boldsymbol{\upsilon }}}{\int }_{{{\boldsymbol{V}}}_{2}}^{{{\boldsymbol{V}}}_{1}}{\boldsymbol{I}}({\boldsymbol{V}}){\boldsymbol{dV}},$$

where *∆V* is a voltage range between the electrodes, *m* is a mass of the active material in a single electrode (g), ν is a scan rate (mV s^−1^). The mass density of the used SWCNT films is 5.1 µg cm^−2^ ^21^. The calculated total capacitance of *C* = 105 μF and specific capacitance of the cell was *C*_sp_ = 82 F g^−1^ or 94 µF cm^−2^. Here, the areal capacitance *(C*_*A*_) was calculated using SWCNT electrode area *(A)* of 1, 5 × 1, 5 cm^2^ and eqns (2) and (3):

2 $${\boldsymbol{C}}=\frac{{{\boldsymbol{C}}}_{{\boldsymbol{sp}}}{\boldsymbol{m}}}{4},$$

3 $${{\boldsymbol{C}}}_{{\boldsymbol{A}}}=\frac{2{\boldsymbol{C}}}{{\boldsymbol{A}}}$$

The GCD curves, shown in Fig. 5B, demonstrate symmetrical behavior and reflect good performance at different DC currents up to 25 A/g throughout the entire potential range. Additionally, the two-electrode setup was tested for durability under 20 000 CV cycles. The results, presented in Fig. 5C, show excellent reliability of the cell. The specific capacitance retained 96% of its initial value. In order to compare performance of the used materials a two-electrode cell supercapacitor with the commercial separator (25 μm thick Celgard separator purchased from MTI Corporation) was evaluated. As shown in Fig. 5D, the Nyquist plots display at the high-frequency region semi-circles of a similar size for both separators and these are attributed to charge transfer processes on the electrode surfaces^32^. Moreover, the straight lines observed in the low-frequency region represent ion diffusion in the system and the more vertical line suggest improved ion transfer trough the BNNT. The extracted equivalent series resistance (ESR) of the supercapacitor assembled in a test cell with BNNT separator (ESR = 4.6 Ω) is comparable to cell assembled with the commercial one (ESR value of 5.9 Ω).

**Figure 6:**
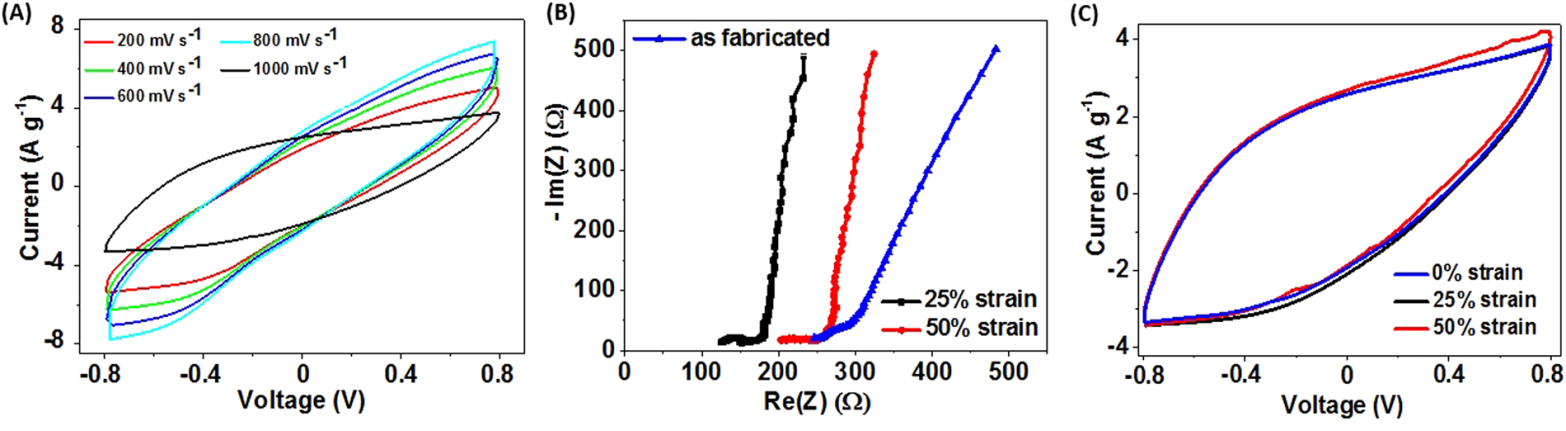
Stretchable supercapacitor characteristics. (**A**) CVs at different scan rates, (**B**) EIS spectra of the as-fabricated supercapacitor (blue) after 100 stretching cycles under 25% (black) strain with inset showing ESR of PVA/H2SO4 used for stretchable supercapacitor, 50% (red) elongation; (**C**) CVs of as-fabricated SSC device (blue), 25% (black) and 50% (red) strain after 1000 stretching cycles.

After the material examination, we characterized the stretchable supercapacitor (SSC) prototype in a two-electrode configuration. Figure 6A demonstrates cyclic voltammograms of the fabricated SSC prototype according to the scheme presented in Fig. 2, at different scan rates. It is worth noting that for the stretchable prototype lower currents and higher impedance obtained in comparison with the test cell. The supercapacitor was stretched up to 25% and 50% strain values for 1000 cycles. CVs at different scan rates and EIS spectra in different stretched positions were measured and shown in Fig. 6C and B, respectively. The fabricated SSC could withstand only one stretching cycle with a 55% strain applied due to the limitation in the pdms substrate mechanical properties. For the as-fabricated stretchable prototype specific capacitance reached (*C*_sp_) 7.7 F g^−1^ and total device capacitance of *C* = 20 μF. Increasing the strain to 50% we observed an increase in the current (Fig. 6C). The value of specific capacitance also increase up to 8.4 F g^−1^ due to better electrolyte diffusion while stretching^23^. As it is shown in Fig. 6B, the lowest ESR value of 125 Ω was measured by means of EIS after 1000 cycles at 25% strain, which slightly increased to 200 Ω after the 50% strain. This value is still lower than ESR = 250 Ω of the as-fabricated device. It is also noteworthy that the changes in the low-frequency region line in the impedance spectra suggest improved ion transfer with the stretching. Another important finding is that the ESR value of the all-nanotube SSC prototype was two orders of magnitude lower compared to the stretchable supercapacitors with PVA/H_2_SO_4_ separator of the similar stretchable configuration (ESR = 15 kΩ). Such a high difference in the ESR yields in a hundred times higher power density of the SSC with the BNNT separator. Additionally, the ESR and capacitance values obtained for the SSC is lower than that of two-electrode test cell supercapacitor, as the test cell electrodes are made of stainless steel, which affects the charge transfer across thr electrodes. It is worth mentioning that for a stretchable supercapacitor the value of the total device thickness (*h*) was around 1100 μm, including the PDMS substrates, and less than 100 μm excluding it, with the electrode area of the device is 2 × 1 cm^2^. Volumetric capacitance C_Vol_ of the device was calculated according to the eqn (4)^33^:

4 $$\,{{\boldsymbol{C}}}_{{\boldsymbol{Vol}}}=\frac{{\boldsymbol{C}}}{{\boldsymbol{A}}\,\cdot {\boldsymbol{h}}}.$$

According to this formula the as-fabricated SSC exhibits the volumetric capacitance of 0.4 mF cm^−3^. As it was mentioned, the SSC device was stretched up to 50% strain without any significant change in the electrochemical performance. It can be seen from Fig. 6C, increasing the strain results in a small increase in the current and consequently in the volumetric capacitance from 0.4 to 0.5 mF cm^−3^.

However, our SSC could withstand only one stretching cycle at 55%, which was explained by appearance of micro cracks in the BNNT separator layer and leading to the short circuit between the SWCNT electrodes. The size of cracks in the BNNT layer was over 10 μm, which is critical for the device performance. Increase in values of volumetric capacitance can be explained by the effect of better electrolyte diffusion and electron transport through the layers due to stretching of the electrodes with the deposited gel electrolyte, which was also discussed in our previous work^22^. Table 1 summarises all calculated values of the as-fabricated SSC device at 0% strain applied and after maximum strain of 50% applied for 1000 stretching/releasing cycles. In order to provide full characterization of the SSC, volumetric energy densities in both stretched and as fabricated conditions were calculated using eqn (5)^5,34^:

5 $${\boldsymbol{W}}=\,\frac{{\bf{0}}.{\bf{5}}\,{{\boldsymbol{C}}}_{{\boldsymbol{sp}}}\,{\boldsymbol{m}}\,{({\rm{\Delta }}{\boldsymbol{V}})}^{2}}{{\bf{4}}\cdot {\bf{3600}}\cdot {\bf{3}}{\boldsymbol{Vol}}},$$

where *Vol* is the total volume of the device, including both electrodes, separator and substrates volumes. Values of the volumetric capacitance and volumetric energy density for supercapacitors assembled in the test cell were calculated and compared with respective values recalculated for the SSC prototype (excluding the thickness of the PDMS substrate) and are also presented in Table 1. As can be seen, the values of volumetric capacitance for supercapacitors assembled in the two-electrode cell configuration with the commercial separator and with BNNTs separator were calculated to be 7.9 and 8.5 mF cm^−3^, respectively. For the SSC prototype at the initially relaxed state this value reaches 0.9 mF cm^−3^ while after 1000 cycles of stretching to 50% strain and almost no degradation was observed with slightly increased value of 1.0 mF cm^−3^.

**Table 1:** Parameters, calculated for all-nanotube supercapacitors: two-electrode cell assembly and stretchable prototype.

Type of the device assembly	Specific capacitance, C_sp_ (F g^−1^)	Areal capacitance, C_A_ (µF cm^−2^)	Volumetric capacitance, C_Vol_ (mF cm^−3^)	Volumetric energy density, W (mW h cm^−3^)	Volumetric power density, P (mW cm^−3^)	Equivalent series resistance, ESR (Ω)
	Two-electrode cell					
Commercial separator	76	68	7.9	3.5	4800	5.9
BNNT separator	82	73	8.5	3.7	6200	4.6
	Stretchable prototype with BNNT separator					
As fabricated	7.7	19	0.9	0.09	32	250
After 1000 cycles of 50% strain	8.4	21	1.0	0.10	40	200

In addition to the volumetric energy density, power density P (W cm^**−**3^) of the supercapacitor is also important for the practical applications and could be calculated according to the following eqn:

6 $${\boldsymbol{P}}=\frac{{\rm{\Delta }}{{\boldsymbol{V}}}^{2}}{4\,{\boldsymbol{ESR}}\cdot {\boldsymbol{Vol}}}.$$

The energy density of 3.7 mW h cm^**−**3^ at the power density of 6200 mW cm^**−**3^ were obtained for the two-electrodes cell with the BNNTs separator, which is higher than the energy density of 3.5 mW h cm^**−**3^ at the power density 4800 mW cm^**−**3^ for the test cell with the commercial separator. The difference can be attributed to slightly lower ESR and smaller thickness of the BNNTs separator. The volumetric energy density for the fabricated and stretched to 50% strain prototype where 0.09 and 0.10 mWh cm^**−**3^, respectively, with improved values of volumetric power density from 32 mW cm^**−**3^ to 40 mW cm^**−**3^ after the stretching. Such impressive difference in the volumetric energy and power densities values can be explained by lower values of specific capacitances of SSC and smaller operating voltage window. For further increase of energy density values, the voltage range of the SWCNT electrodes in an aqueous electrolyte can be increased by purifying the SWCNTs form Fe catalyst particles. On the other hand, alternative electrolytes, such as organic solvents or ionic liquids with larger electrochemical window, can be used to increase the energy density values. However, the values of volumetric power densities obtained in this work can be compared with the reported in the literature^33,35,36^ for flexible and stretchable all-solid-state energy storage devices based on similar classes of nanomaterials.

## Conclusions

High-performance, stable, low equivalent series resistance, all-nanotube stretchable supercapacitor based on single-walled carbon nanotube film electrodes and boron nitride nanotube separator have been successfully fabricated. The fabrication process is very simple, fast and based on dry transfer and airbrush techniques. The SSC prototype indicates the possibility and advantages of using the SWCNT electrodes and BNNT separator as reliable components in stretchable energy storage devices. The fabricated two-electrode test cell with the materials mentioned above demonstrates excellent stability after 20 000 electrochemical charging/discharging cycles, and EDLC behaviour and retains 96% of its initial capacitance of 82 F g^−1^. The ESR value of 4.6 Ω of such supercapacitor with BNNTs separator layer is lower than the value for the device assembled with commercial one (5.9 Ω). Moreover, a stretchable supercapacitor fabricated with the same materials withstands at least 1000 cycles of 50% strain with a slight improvement in the performance. The volumetric capacitance of the SSC is 0.4 mF cm^−3^ initially including thickness of the PDMS substrates into the volume of the device, and has the maximum value of 0.5 mF cm^−3^ after stretching to 50% strain. In addition, SSC has superior values of volumetric power density, which improve from 32 mW cm^−3^ to 40 mW cm^−3^ after the stretching. The ESR value for SSC reached 200 Ω after the stretching. Remarkably, the BNNT separator of only 0.5 µm thickness ensures reliable short circuit protection and low ESR of the SSC. These exciting findings demonstrate that SCs fabricated with the method used in this research hold a great promise for use as high-performance stretchable energy storage devices in the future.
